# Appropriate vitamin D loading regimen for patients with advanced lung cancer

**DOI:** 10.1186/s12937-016-0203-8

**Published:** 2016-10-06

**Authors:** L. John Hoffer, Line Robitaille, Nelda Swinton, Jason Agulnik, Victor Cohen, David Small, Carmela Pepe, Shaun Eintracht

**Affiliations:** 1 Lady Davis Institute for Medical Research, McGill University and Jewish General Hospital, 3755 Cote Sainte Catherine, Montreal, QC H3T 1E2 Canada; 2 Pulmonary Oncology Peter Brojde Lung Cancer Centre, Jewish General Hospital, Montreal, Canada; 3 Department of Diagnostic Medicine, Jewish General Hospital, Montreal, Canada

## Abstract

**Background:**

Most patients attending cancer clinics have hypovitaminosis D. Correcting or preventing this abnormal condition could mitigate the emotional and physical complications of their disease, but clinical trials of vitamin D therapy in this setting are hindered by the unavailability of safe, effective and practical loading dose regimens.

**Methods:**

In this single arm open-label pharmacokinetic trial, outpatients with advanced lung cancer consumed 20,000 IU vitamin D daily with the largest meal of the day for 14 days followed by 10,000 IU per day for a further 7 days. Plasma concentrations of 25-hydroxyvitamin D [25(OH)D], parathyroid hormone, calcium, vitamin C and C-reactive protein were measured on protocol days 0, 14 and 21, and serum vitamin D binding protein (VDBP) concentrations on days 0 and 21. As a secondary objective, preliminary information was obtained regarding clinical effects of rapid vitamin D loading on mood and symptoms by administering appropriate questionnaires two times at baseline and after 14 and 21 days of vitamin D therapy.

**Results:**

Of the 91 patients enrolled in the study, 85 % had hypovitaminosis D and 41 % had hypovitaminosis C. Plasma VDBP concentrations were in the normal range. The vitamin D load increased the average plasma 25(OH)D concentration to 116 ± 34 nmol/L (mean ± SD); the median concentration was 122 nmol/L (interquartile range 103–134); VDBP concentrations did not change. Final plasma 25(OH)D concentrations were subnormal (<75 nmol/L) for 13 % of the patients and sub-target (<120 nmol/L) for 44 % of them. In most cases, subnormal and sub-target 25(OH)D concentrations were attributable to obesity and/or a low baseline 25(OH)D concentration. Mood and symptom scores did not change significantly throughout the 3-week protocol.

**Conclusion:**

Hypovitaminosis D and C are very common in outpatients with advanced lung cancer. A vitamin D load of 20,000 IU per day for 14 days failed to achieve the target concentration in 44 % of the participants in this trial. These results suggest that a loading dose of 30,000 IU per day for 14 days would be safe and effective for patients who are obese or at risk of severe hypovitaminosis D. The preliminary nature of the study design, and the failure to achieve target 25(OH)D concentrations for a large proportion of the patients, do not allow any firm conclusion about the clinical effects of correcting hypovitaminosis D in this patient population. Nevertheless, no evidence was obtained that partial correction of hypovitaminosis D greatly improved mood, reduced distress or relieved cancer-related symptoms. This trial was registered at clinicaltrials.gov as NCT01631526.

## Background

Hypovitaminosis D–a plasma 25-hydroxyvitamin D [25(OH)D] concentration < 75 nmol/L [1]–is present in the great majority of patients attending cancer clinics [2–4], but its clinical consequences are unknown. There is general agreement that biochemical vitamin D deficiency [plasma 25(OH)D < 25 nmol/L] requires prevention and treatment, but the benefits of correcting vitamin D insufficiency [plasma 25(OH)D between 25 and 75 nmol/L] are uncertain and a topic of active clinical investigation [1, 5–7].

Correcting subclinical vitamin D deficiency in patients with advanced cancer, including lung cancer, could mitigate some of the emotional and physical complications of their disease, including pain [8, 9] and certain side effects of cytotoxic chemotherapy [10], since vitamin D has important functions in relation to brain metabolism [11–13], muscle function [14–16] and immunity [16–21].

Clinical trials of vitamin D therapy in actively progressing diseases like lung cancer are hindered by the unavailability of safe, effective and practical loading dose regimens. The primary objective of this study was to test the ability of a simple vitamin D loading regimen to rapidly attain a target 25(OH)D concentration in every patient. The resulting plasma 25(OH)D concentrations were interpreted in the context of a systematic review of the available data pertaining to rapid vitamin D loading in a variety of patient populations. As a secondary objective, we tested the ability of three questionnaires to measure mood, well-being, and cancer-related symptoms in the context of vitamin D loading. More than one subclinical micronutrient deficiency can occur in a patient with cancer [22]. To explore this possibility, we measured plasma vitamin C concentrations in the study participants.

## Methods

### Justification of the loading dose protocol

#### A loading dose is necessary

Exploratory clinical trials in patients with actively progressing disease have to be implemented promptly and be of relatively short duration, a time constraint that rules out simple maintenance therapy which requires months to attain a steady-state target plasma 25(OH)D concentration [23, 24].

#### Target 25(OH)D concentration

A variety of biological and observational data suggest that 25(OH)D concentrations in the range of 100–130 nmol/L are safe and potentially optimal for human health [6, 7, 25], hence a promising target in clinical trials with non-bone-related endpoints. A suitable loading dose regimen will bring the plasma 25(OH)D concentration of every patient–not merely the group average–into the target range. The target plasma 25(OH)D concentration in this study was 120 nmol/L for every patient.

#### Avoid very large individual doses

Vitamin D_3_ (cholecalciferol) is the precursor of the active form of the vitamin, 1,25-dihydroxyvitamin D, but cholecalciferol itself has some biological activity and when administered in unphysiologically large individual doses could engender adverse effects consequent to variable storage and release of the unmetabolized vitamin [26–30]. We reasoned that a safe and reliable loading regimen should avoid daily doses much greater than the maximum rate of sunlight-induced endogenous vitamin D synthesis, approximately 10,000–20,000 IU per day [30–32].

#### Bioavailability

Fat-soluble vitamins like vitamin D are better absorbed when consumed with fat-containing meals [33–35].

#### Practicality

High quality pharmaceutical vitamin D formulations in a dose of 10,000 IU per tablet are widely available.

#### Safety

The loading dose had to be conservative enough to mitigate concerns about potential toxicity among treating oncologists and patients considering participation in a clinical trial.

### Details of the loading dose protocol

Study participants took 20,000 IU vitamin D (two tablets) daily for 14 days (total dose 280,000 IU) followed by 10,000 IU per day for a further 7 days (for a total dose of 350,000 IU over the entire 3-week study period). Study participants were instructed to take their daily vitamin D dose with the largest meal of the day rather than on an empty stomach. The daily maintenance dose of 10,000 IU was chosen because of evidence that it is safe to use in a clinical trial [32, 36] and can sustain plasma 25(OH)D concentrations in the vicinity of 120 nmol/L for almost every patient [6].

### Vitamin D binding protein

Approximately 90 % of the 25(OH)D measured in a serum or plasma sample is bound to the vitamin D binding protein (VDBP) [30, 37]. It is unclear how much changes in plasma VDBP concentrations affect measured plasma 25(OH)D concentrations or biological effects in people who are acutely sick, since systemic inflammation expands the extravascular space and redistributes large proteins into the extracellular space, potentially confounding the analysis of kinetic studies of vitamin D concentration and effect [30, 38–40]. To address this possibility we measured plasma VDBP concentrations at baseline and after 3 weeks of vitamin D therapy.

### Outcome measures

The primary pharmacokinetic outcome was plasma 25(OH)D concentrations measured at baseline, after 14 days of vitamin D loading (20,000 IU per day), and after a further 7 days of the maintenance dose of 10,000 IU per day. Secondary outcome measures were mood, distress, and cancer-related symptoms evaluated at the same time points. The prevalence of hypovitaminosis D and C in this patient population were documented as well as their relationship to certain biologically relevant factors, including systemic inflammation. In addition to parathyroid hormone concentrations, standard information obtained for clinical purposes was also analyzed, specifically including serum calcium and C-reactive protein.

### Participants

Mentally competent ambulatory outpatients of any age > 18 years with advanced lung cancer were eligible to participate whether or not they were receiving specific anti-cancer therapy, with the following exceptions: current use of any vitamin D supplement equivalent to > 1000 IU per day, current therapy with any dose of calcitriol, a recent history of extensive sunlight exposure (>30 min of summer sunlight exposure more than 5 days per week), non-fluency in French or English and no capable neutral translator available, current diagnosis of primary hyperparathyroidism, existing nephrocalcinosis, hypercalcemia, current or suspected tuberculosis, histoplasmosis, sarcoidosis or other granulomatous disease, pregnancy, or anticipated death within 2 months.

### Study site and details

The Pulmonary Oncology Peter Brojde Lung Cancer Centre accepts approximately 180 new referrals each year; approximately 375 patients are being followed at any time. Approximately 80 % of newly referred patients have advanced lung cancer treated with palliative chemotherapy, radiotherapy and supportive care. The vitamin D used in the trial was provided to patients by the hospital’s research pharmacy in a labeled bottle containing 35 tablets of 10,000 IU (Laboratoire Riva Inc, Blainville QC, Canada; DIN 00821772). The patients were followed by the dietitian (NS) who administered the questionnaires and made pill counts at each visit to verify compliance. The accrual goal of the study was completion of the 3-week protocol by 80 patients (“completed study group”). The baseline data for patients who enrolled in the study but did not complete the entire protocol (“initial study group”) were used to determine the frequency and clinical correlates of hypovitaminosis C and D. The study protocol was approved by the Research Ethics Committee of the Jewish General Hospital and registered at clinicaltrials.gov (NCT 01631526).

### Research analytical measurements

#### Plasma 25(OH)D, VDBP, vitamin C and other concentrations

Total 25-hydroxyvitamin D was measured by competitive electrochemiluminescence binding (Roche, Laval, QC) on a Cobas e 602 module (Roche, Laval, QC). The assay uses a vitamin D binding protein (VDBP), which binds both 25-hydroxyvitamin D2 and 25-hydroxyvitamin D3, as a capture protein. At a concentration of 21 nmol/L the assay has intra-assay and inter-assay coefficients of variation of 6.8 and 13.1 % respectively; and at a concentration of 174 nmol/L, 2.2 and 3.4 % respectively. VDBP was measured in plasma by means of a solid phase enzyme immunoassay using the Quantikine Human Vitamin D Binding Protein kit (R&D Systems, Minneapolis, MN). Patient samples were stored at < −20 ° C prior to analysis and analyzed in batches using kits of the same lot number. The coefficients of variation of intra-assay and inter-assay concentrations were 4.6 and 9.1 % respectively. For internal validation, we measured plasma VDBP in 22 healthy volunteers not receiving any medication and taking either no vitamin D supplements or < 1000 IU vitamin D daily. The values obtained for these volunteers (average 196 μg/mL, SD 79, range 74–318) fell within the reference interval provided by the manufacturer. Intact parathyroid hormone (reference interval 10–70 ng/L) was measured in plasma by electrochemiluminescence immunoassay on a Cobas e 602 module (Roche, Laval, QC). Plasma C-reactive protein (reference interval 1–10 mg/L) was measured by latex particle enhanced immunoturbidimetry on a Cobas e 502 module (Roche, Laval, QC). Plasma vitamin C was measured as described previously [41].

### Mood and symptom assessment

#### POMS-B

The Profile of Mood States™ (POMS™) is a widely used 65 item questionnaire that measures mood in healthy, physically ill and psychiatric populations; the instrument generates a total mood disturbance score [42, 43]. Briefer versions of the POMS™ have been developed to accommodate the limited reserve of physically ill patients and found to be practical and valid [42, 44, 45]. The 30-item POMS™-B was chosen as the most appropriate instrument to assess the mood of cancer patients because it is a validated, extensively used, broad spectrum tool that can be conveniently and quickly administered even to sick, hospitalized patients [46].

#### Distress thermometer

This simple, validated one-item measure of psychological distress asks the patient to indicate their level of distress alongside an image of a thermometer [47–49]. The DT is formally recommended by several clinical organizations as a valid, easy to use and simple tool for detecting distress in people with cancer [47].

#### Revised Edmonton Symptom Assessment System (ESAS-r)

This very widely used 9-question self-report symptom intensity tool is used to assess the intensity of nine common symptoms experienced by cancer patients. The items scored by this questionnaire are: pain, tiredness, drowsiness, nausea, lack of appetite, shortness of breath, anxiety, depression and well-being [50].

### Systematic review

In order to interpret our observations and explore whether people with advanced lung cancer differ importantly in their response to vitamin D loading from other people, we carried out a systematic review of the literature pertaining to the effects of vitamin D loading regimens on plasma or serum 25(OH)D concentrations in the 3 to 21-day time period in adults. Pertinent articles were identified by studying and up-dating three published systematic reviews of the kinetics of high-dose vitamin D conversion to 25(OH)D [51–53] that included data in the 3 to 21-day period. This process identified six articles [54–59]. Ten more pertinent articles were identified by studying the references cited in these reviews and updating the searches [60–69]. One otherwise pertinent study [70] was excluded because it provided incremental, but no baseline data. The search process identified 16 articles that reported 24 different kinetic studies that included plasma 25(OH)D measurements at early time points after large oral doses of vitamin D.

### Statistical methods

Statistical analyses were performed using GraphPad Prism version 5.01 (GraphPad Software Inc., San Diego, Ca). Non-parametric tests were used in all cases. Correlation analyses between baseline metabolites were done using Spearman coefficients. Wilcoxon matched pairs tests were used to verify differences between pre-baseline and baseline results. The Friedman test and Dunn’s post test were performed to detect differences between baseline and post-treatment results. The significant level of *P* < 0.05 was adopted in all analyses.

## Results

**Fig. 1:**
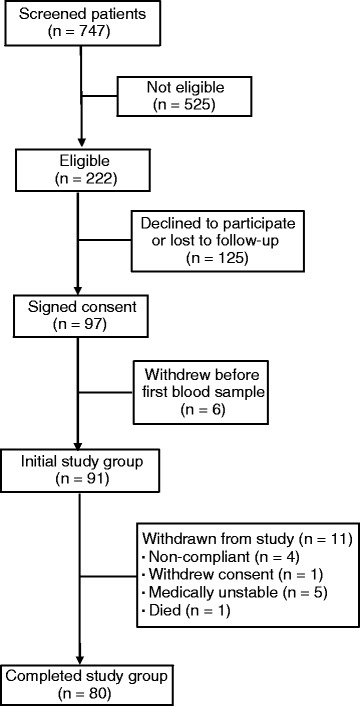
Flow diagram

The first participant was enrolled on June 14, 2012 and the final, eightieth one completed the protocol on November 3, 2015. The study flow diagram is shown in Fig. 1. During the approximately 3.5-year duration of the study, 747 patients were screened for eligibility, of whom 525 were ineligible (reasons indicated in Table 1). Of the 222 eligible patients, 125 were uninterested in participating or lost in the rapid flux of patients in and out of the clinic. Six patients who agreed to participate withdrew or had to be withdrawn before the protocol commenced, leaving 91 people who began the study (initial study group). Eleven participants were withdrawn before the protocol could be completed (reasons indicated in the study flow diagram, Fig. 1), leaving 80 patients who completed the protocol. The baseline characteristics of the 91 patients who commenced the study (initial study group, *n* = 91) and the 80 who completed it (study completed group, *n* = 80) are shown in Table 2. The characteristics of the study-completed group were closely similar to those in the initial study group. Although we cannot rule the possibility out, in almost every case the reasons for ineligibility do not suggest that the nonparticipants were clinically or metabolically very different from the people who enrolled in and completed the study. No patient was prescribed calcitriol or suffered from renal insufficiency.

**Table 1:** Reasons for ineligibility

Reason	Number	Percent^a^
Early stage lung cancer	137	26.1
Clinically unstable	118	22.5
Vitamin D usage	84	16.0
Came for second opinion	55	10.5
Language barrier	37	7.0
On another study	23	4.4
Not lung cancer	29	5.5
Mentally incompetent	14	2.7
High serum calcium	14	2.7
Unreliable	7	1.3
Other	7	1.3

Of 747 patients evaluated, 525 were ineligible to participate

^a^Percent of ineligible patients. Some patients had more than one exclusion criterion; only the first reason or main condition is noted

**Table 2:** Baseline characteristics

Variable	Initial study group (*n* = 91)	Study completed group (*n* = 80)
Age (y)	63.0 ± 11.2	63.3 ± 11.4
Male sex (%)	49	51
Smoker (%)	32	33
Weight loss history (%)	52	51
Body weight (kg)	71.9 ± 14.6	72.6 ± 14.9
BMI (kg/m^2^)	26.1 ± 4.4	26.1 ± 4.6
Blood hemoglobin (g/L)	121 ± 19	121 ± 19
Serum albumin (g/L)	39 ± 4	39 ± 4
Plasma 25(OH)D (nmol/L)	47.6 ± 28.1	48.2 ± 28.3
Subnormal (%)	85	84
Deficient (%)	26	25
Plasma total vitamin C (μmol/L)	41.5 ± 27.6	42.5 ± 27.5
Subnormal (%)	41	40
Plasma vit D binding protein (μg/mL)	228 ± 63	230 ± 64
Serum calcium (mmol/L)	2.34 ± 0.10	2.33 ± 0.09
Plasma parathyroid hormone (ng/L)	48.8 ± 25.1	48.7 ± 23.7
Plasma C-reactive protein (mg/L)	21.3 ± 38.0	20.9 ± 36.8
Total mood disturbance score	10.7 ± 17.9	10.0 ± 17.1
Distress Thermometer score	2.1 ± 2.6	1.9 ± 2.2
ESAS-r	20.6 ± 14.4	20.3 ± 14.0

Data are presented as mean ± SD. Reference intervals are as follows: hemoglobin (men 140–175 g/L, women 120–152 g/L), albumin (35–51 g/L), C-reactive protein (<10 mg/L), parathyroid hormone (10–70 ng/L), calcium (2.12–2.62 mmol/L), total vitamin C (>28.3 μmol/L), 25(OH)D (75–250 nmol/L) and deficient level (<25 nmol/L), vitamin D binding protein (43–415 μg/mL). The total mood disturbance score ranges from −20 to 100, the Distress Thermometer score ranges from 0 to 10. and the ESAS-r (revised Edmonton Symptom Assessment System) ranges from 0 to 100

Percent smoker includes individuals who quit less than 1 year before enrollment

Percent weight loss history refers to a history of involuntary weight loss at onset of study

### Baseline data

As shown in Table 2, 85 % of the patients in the initial study group (*n* = 91) had hypovitaminosis D; 26 % of them had previously undiagnosed biochemical vitamin D deficiency [plasma 25(OH)D concentration < 25 nmol/L]. Plasma VDBP concentrations were in the normal range. Plasma 25(OH)D was strongly correlated with plasma VDBP (*P* = 0.0015), although the predictive value of the association was low (Spearman *r* = 0.33). The usual negative correlation between plasma 25(OH)D and parathyroid hormone concentrations was observed (*P* = 0.002, *r* = −0.32). Hypovitaminosis C was present in 41 % of the patients. There was a strong negative correlation between plasma vitamin C and C-reactive protein, an indicator of systemic inflammation (*P* = 0.0068, *r* = −0.28), but no correlation between plasma VDBP and C-reactive protein nor between 25(OH)D and C-reactive protein.

### Effects of the loading dose on plasma 25(OH)D and VDBP concentrations

**Fig. 2:**
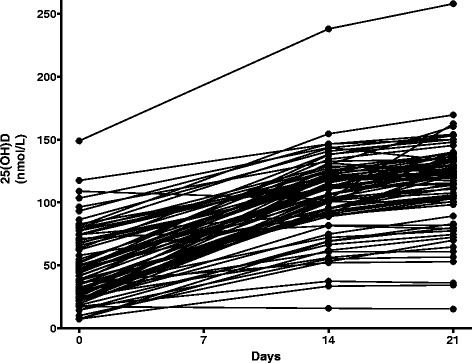
Individual plasma 25(OH)D concentrations before and after 14 and 21 days of oral vitamin D

**Fig. 3:**
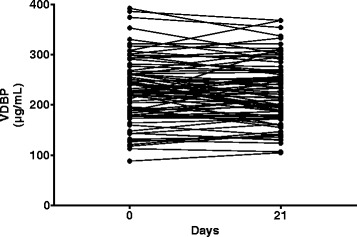
Individual plasma vitamin D binding protein concentrations before and after 21 days of oral vitamin D

As shown in Table 3 and Fig. 2, the vitamin D loading regimen increased plasma 25(OH)D concentrations dramatically within 14 days, with a further small increase by day 21. The average final plasma 25(OH)D concentration was 116 ± 34 nmol/L (mean ± SD); the median concentration was 122 nmol/L (interquartile range 103–134). Plasma parathyroid hormone levels decreased after vitamin D therapy (Table 3), the expected biological response to correcting vitamin D deficiency. Plasma VDBP concentrations remained constant and normal during the protocol (Table 3 and Fig. 3).

**Table 3:** Effects of vitamin D therapy in study completed group

Variable	Pre-baseline	Baseline	14 days (280,000 IU)	21 days (350,000 IU)
Plasma 25(OH)D (nmol/L)		48.2 ± 28.3	105.5 ± 31.7^a^	116.2 ± 34.3^a^
< 120 nmol/L (%)		99	69	44
< 75 nmol/L (%)		84	18	13
< 25 nmol/L (%)		25	1	1
Plasma total vitamin C (μmol/L)		42.5 ± 27.5	42.9 ± 25.4	42.0 ± 22.8
Subnormal (%)		40	36	30
Plasma vit D binding protein (μg/mL)		230 ± 64	-	227 ± 63
Serum calcium (mmol/L)		2.33 ± 0.09	2.37 ± 0.11	2.37 ± 0.10
Plasma parathyroid hormone (ng/L)		48.7 ± 23.7	39.7 ± 18.9^b^	38.7 ± 18.2^b^
Plasma C-reactive protein (mg/L)		20.9 ± 36.8	17.0 ± 22.1	16.7 ± 22.0
Total mood disturbance score	10.4 ± 17.6	10.0 ± 17.1	7.8 ± 17.0	9.4 ± 19.0
Distress Thermometer score	2.1 ± 2.4	1.9 ± 2.2	1.7 ± 2.3	1.6 ± 2.2
ESAS-r	20.3 ± 15.3	20.3 ± 14.0	17.4 ± 13.6	17.7 ± 16.0^c^

Mean ± SD. Details of variables and reference intervals as in Table 2. Superscripts 1, 2 and 3 indicate Dunn’s post-test comparisons following the Friedman test

^a^ Different from baseline and from each other (*P* < 0.0001)

^b^ Different from baseline but not from each other (*P* < 0.0001)

^c^ Different from baseline (*P* = 0.012)

**Fig. 4:**
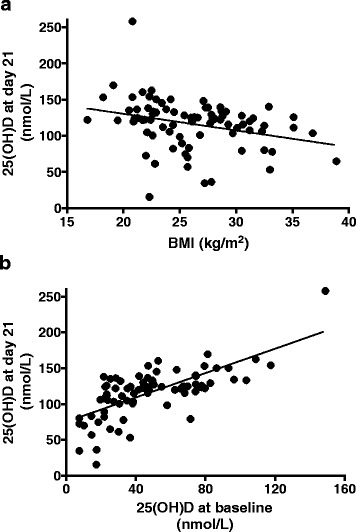
Relationships between body mass index (**a**) and baseline 25(OH)D concentration (**b**) with the final 25(OH)D concentration. Spearman *r* values: −0.33 (*P* = 0.0032) and 0.69 (*P* < 0.0001) respectively. The linear regression lines are indicated

Plasma 25(OH)D concentrations on day 21 were strongly associated with body mass index (BMI) and baseline 25(OH)D concentration, as shown in Fig. 4. Final plasma 25(OH)D concentrations were < 120 nmol/L for 44 % of the participants (35 patients, 26 of whom had a BMI > 30 kg/m^2^ or baseline 25(OH)D < 30 nmol/L. Ten participants had a final 25(OH)D concentration < 75 nmol/L; nine of them had a very low baseline 25(OH)D concentration, and two had a BMI > 30 kg/m^2^.

The results of the systematic review of articles reporting plasma 25(OH)D concentrations shortly after high-dose vitamin D administration are displayed in Table 4.

**Table 4:** Early time-point effects of oral vitamin D loading on plasma 25(OH)D concentrations of adults

First author, year [reference]	Number	Study population	BMI	Intervention	Time point	Mean 25(OH)D concentration	Range or SD
Weisman et al., 1986 [60]	13	Elderly	n.a.	100,000 IU in one dose	Baseline	29	
					14 days	88	SD 14
Ilahi et al., 2008 [54]	30	Healthy volunteers	27	100,00 IU in one dose	Baseline	27	
					7–21 days^a^	105	SD 20
Romagnoli, 2008 [56]	8	Elderly nursing home	n.a.	300,000 IU in one dose	Baseline	33	
					3 days	120	n.a
					7 days	128	
					30 days	152	
Martineau et al., 2009 [55]	56	Normal volunteers	n.a.	100,000 IU vitamin D2 in one dose	Baseline	34^b^	
					7 days	102	50–140
”	11	Active tuberculosis	n.a.	100,000 IU vitamin D2 in one dose	Baseline	23^b^	
					7 days	133	70–250
Cipriani et al., 2010 [61]	48	Healthy volunteers	24	600,000 IU in one dose	Baseline	35	
					3 days	193	SD ~68
					15 days	193	
					30 days	155	
Amrein et al., 2011 [57]	12	Critically ill	29	540,000 IU in one dose	Baseline	35	
					3 days	88	n.a.
					7 days	96	
Rossini et al., 2012 [58]	13	Elderly	29	100,000 IU in one dose	Baseline	66	
					7 days	85	SD ~20
					14 days	83	
					30 days	84	
”	12	”	29	300,000 IU in one dose	Baseline	64	
					7 days	96	SD ~20
					14 days	95	
					30 days	86	
”	12	”	29	600,000 IU in one dose	Baseline	54	
					7 days	156	SD ~30
					14 days	152	
					30 days	129	
Grossmann et al., 2012 [59]	15	Adults with cystic fibrosis and acute illness	18.5	250,000 IU in one dose	Baseline	76	
					7 days	145	SD 34
Roth et al., 2012 [62]	34	Non-pregnant women	n.a.	70,000 IU in one dose	Baseline	54	
					7 days	79	~45–125
					14 days	75	~40–140
”	27	Pregnant women	n.a.	70,000 IU in one dose	Baseline	39	
					7 days	67	~45–100
					14 days	71	~50–120
De Jong et al., 2013 [63]	14	Elderly acute hip fracture	n.a.	150,000 IU as 50,000 IU/day for 3 days	Baseline	30	
					7 days	81	47–108
”	54	”	n.a.	350,000 IU as 50,000 IU/day for 7 days	Baseline	31	
					7 days	131	86–243
Drincic et al., 2013 [64]	20	Obese	38	10,000 IU per day	Baseline	58	
					7 days	80	n.a.
					21 days	106	
Cantor, 2014 [65]	18	Internal medicine patients	n.a	356,000 IU as 300,00 IU followed by 4000 IU/day	Baseline	22	
					14 days	102	50–202
”	32	”	n.a.	206,000 IU as150,000 IU followed by 4000 IU/day	Baseline	55	
					14 days	108	58–185
Kearns et al., 2015 [66]	14	Healthy volunteers	24	250,000 IU in one dose	Baseline	41	
					5 days	102	70–149
Oliveri et al., 2015 [67]	11	Healthy volunteers	22	100,000 IU in one dose	Baseline	41	SD 12
					7 days	105	
”	11	”	21	100,000 IU vitamin D2 in one dose	Baseline	41	SD 28
					7 days	92	
Rousseau et al., 2015 [68]	29	Healthy volunteers	n.a.	100,000 IU in one dose	Baseline	54	
					7 days	100	64–167
”	20	Acute burn injury	n.a.	100,000 IU in one dose	Baseline	18	
					7 days	48	12–84
Tukvadze et al., 2015 [69]	100	Active tuberculosis	n.a.	50,000 IU three times weekly	Baseline	35	
					14 days	175	n.a.
					28 days	225	
Present study	80	Lung cancer	26	20,000 IU/day for 14 days then 10,000 IU/day	Baseline	48	
					14 days	106	16–238
					21 days	116	15–258

*BMI* body mass index (kg/m^2^). 25(OH)D concentrations are expressed in nmol/L

^a^The concentration peaked on day 7 and remained approximately constant throughout day 21

^b^ Sum of 25(OH)D_2_ and 25(OH)D_3_

### Toxicity

Serum calcium levels remained constant and normal for every patient. As shown in Fig. 2, one patient whose 25(OH)D concentration was unusually high at baseline experienced an increase to 258 nmol/L, marginally higher than the upper value of the reference interval, 250 nmol/L [5].

### Effects of the loading dose on mood, distress and symptoms

The POMS-B, DT and ESAS-r were administered twice, a week apart, before commencing vitamin D therapy, and repeated on protocol days 14 and 21. As shown in Table 3, average mood and symptom scores did not change upon repeat measurement nor did they change significantly throughout the 3-week protocol, despite an indication that cancer symptoms were slightly reduced by week 3 of vitamin D therapy (*P* = 0.012). There was no strong indication of an important effect of vitamin D therapy on any sub-scale of the questionnaires nor of benefit in those patients whose 25(OH)D concentration was very low at baseline and normalized during the 21-day duration of the study.

## Discussion

This study corroborates previous reports that hypovitaminosis D is highly prevalent in outpatients being treated for advanced cancer [2–4] and further demonstrates a high prevalence of hypovitaminosis C. One-quarter of the patients in this study had previously unsuspected biochemical vitamin D deficiency, a diagnosis that calls for immediate therapy. Neither 25(OH)D nor VDBP levels were related to the level of systemic inflammation as indicated by plasma C-reactive protein concentrations. These data indicate that, unlike vitamin C [41], 25(OH)D and VDBP do not behave as negative acute-phase reactants in this patient population.

The vitamin D loading protocol was safe in that no patient experienced a 25(OH)D level significantly greater than normal, even in the small minority of patients whose 25(OH)D level was normal at baseline. No patient experienced hypercalcemia or any toxicity attributable to vitamin D administration. However, the protocol failed to increase plasma 25(OH)D concentrations into the normal range (75 nmol/L or greater) in 13 % of the participants, and it failed to achieve the target of 120 nmol/L for 44 % of the participants. Obesity [24, 71, 72] and a very low baseline plasma 25(OH)D concentration [23] are known to predict a muted 25(OH)D response to vitamin D therapy. Of the ten patients whose final 25(OH)D concentration was < 75 nmol/L at the end of the protocol, nine had a very low baseline 25(OH)D concentration, and two had a BMI > 30 kg/m^2^. Of the 35 patients whose final 25(OH)D concentration was < 120 nmol/L, 74 % of them either had a BMI > 30 kg/m^2^ or baseline 25(OH)D < 30 nmol/L.

Our observations regarding the short-term effect of this vitamin D loading regimen on 25(OH)D concentrations were interpreted by reference to the systemic review of published data displayed in Table 4. The wide heterogeneity of dose regimens and patient types precludes a statistical analysis, but a qualitative pattern is readily discernable and the present data are consistent with it. It appears that people with advanced lung cancer do not differ from other people in their response to vitamin D loading. Obesity and markedly subnormal baseline 25(OH)D concentrations predict a muted 25(OH)D response to vitamin D loading, whereas active tuberculosis (a disease in which vitamin D metabolism can be altered) is associated with very large increases [55, 69], perhaps partly attributable to the diminished body fat store [73] that so often accompanies active tuberculosis [55].

The present data suggest a convenient way to choose an appropriate vitamin D loading dose using readily available formulations. Even while acknowledging the non-linearity of vitamin D kinetics [74] it would be reasonable to routinely prescribe a loading dose 50 % higher than used in this study, namely, 30,000 IU per day for 14 days for every patient or, alternatively, prescribe 20,000 IU per day for 14 days for nonobese patients but 30,000 IU for patients who are obese [71] or predicted to have severe hypovitaminosis D on the basis of no prior use of a vitamin D supplement, limited sunlight exposure, dark skin coloration, gastrointestinal disease, or use of certain medications [24].

As a secondary objective, we tested the reproducibility of the mood and symptom questionnaires to determine whether they are robust instruments in this patient population, and to gain some preliminary notion of whether any large or striking effect on mood and symptoms could be detected. The mood, emotional well-being and symptom scales used in this study proved to be practical and reproducible; they could be used in future clinical trials in this population. It is emphasized that this study was not designed to, nor could it, answer specific questions about the effects of short-term vitamin D replacement on mood and symptoms. For one thing, the dosing protocol fell short of the target 25-hydroxyvitamin D concentration. For another, the study was not blinded. That having been said, rapid partial correction of vitamin D status as the sole nutritional had no obvious major effect on mood, distress or cancer-related symptoms in this study.

Malnutrition is common in patients with advanced cancer despite their commonly normal body mass index [75]. Such patients are at risk of subclinical deficiencies of other micronutrients [22], as was demonstrated in this study with regard to vitamin C. It is reasonable to presume that normal status with regard to all vitamins and essential minerals, not just certain selected ones, will provide the best outcomes. Future controlled clinical trials testing the benefits of correcting micronutrient deficiencies in people with advanced cancer should use a combination of micronutrient supplements that target documented subclinical deficiencies, in addition to optimal standard symptom management.

## Conclusion

Hypovitaminosis D and C are highly prevalent among outpatients with advanced lung cancer. Although the consumption of 20,000 IU vitamin D per day for 14 days failed to achieve the target 25(OH)D concentration of 120 nmol/L for 44 % of the participants, the data suggest that a loading dose of 30,000 IU per day for 14 days would be safe and effective for patients who are obese or have severe hypovitaminosis D at baseline.

## Abbreviations

25(OH)D: 25-hydroxyvitamin D_3_
BMI: Body mass index
DT: Distress thermometer
ESAS-r: Revised Edmonton Symptom Assessment System
IU: International units
POMS-B: Profile of Mood States-B™
VDBP: Vitamin D binding protein
